# Evaluation of clinical and laboratory characteristics of patients with cutaneous sarcoidosis: A single-center retrospective cohort study

**DOI:** 10.3389/fmed.2022.980507

**Published:** 2022-10-10

**Authors:** Katharina Boch, Ewan A. Langan, Detlef Zillikens, Ralf J. Ludwig, Khalaf Kridin

**Affiliations:** ^1^Department of Dermatology, University of Lübeck, Lübeck, Germany; ^2^Manchester Sciences, University of Manchester, Manchester, United Kingdom; ^3^Lübeck Institute of Experimental Dermatology, University of Lübeck, Lübeck, Germany; ^4^Azrieli Faculty of Medicine, Bar-Ilan University, Safed, Israel

**Keywords:** sarcoidosis, inflammation, skin, patient phenotyping, cutaneous sarcoidosis

## Abstract

**Background:**

Cutaneous sarcoidosis is a relatively rare disease whose clinical manifestations include red-brown macules, plaques, papules and subcutaneous nodules. The skin changes may also be restricted to pre-existing scars. Cutaneous sarcoidosis can be associated with systemic organ involvement.

**Objectives:**

Aim of this retrospective study was to longitudinally investigate clinical and laboratory findings in patients with cutaneous sarcoidosis.

**Methods:**

Patients (>18 years) with histologically confirmed cutaneous sarcoidosis between January 2014 and December 2020 were included. Patient demographics, clinical features, laboratory and radiological findings, management, clinical outcomes and co-morbidities associated with cutaneous sarcoidosis were analyzed.

**Results:**

Thirty-seven patients with cutaneous sarcoidosis were identified, of whom 57% were female. The most common clinical phenotype of cutaneous sarcoidosis was papular sarcoidosis (*n* = 16), while plaques and nodules were present in 9 patients. In contrast, subcutaneous (*n* = 1) and scar-associated sarcoidosis (*n* = 1) were rare. Of patients with systemic disease, the cutaneous disease followed, preceded, and coincided with the development of systemic sarcoidosis in 2, 9, and 12 patients, respectively. Levels of soluble interleukin (IL)-2 receptor, angiotensin converting enzyme (ACE), and C-reactive protein (CRP) were elevated, in 76%, 21%, and 50% of the tested patients respectively and predicted systemic involvement. Hypercalcemia was present in 6% of patients. Female sex and younger age (<54 years) were significantly associated with systemic manifestations.

**Conlcusions:**

Cutaneous sarcoidosis was frequently associated with additional systemic involvement, particularly when present in young females. 24 % of patients with cutaneous sarcoidosis developed additional organ involvement during follow-up.

## Introduction

**Figure 1:**
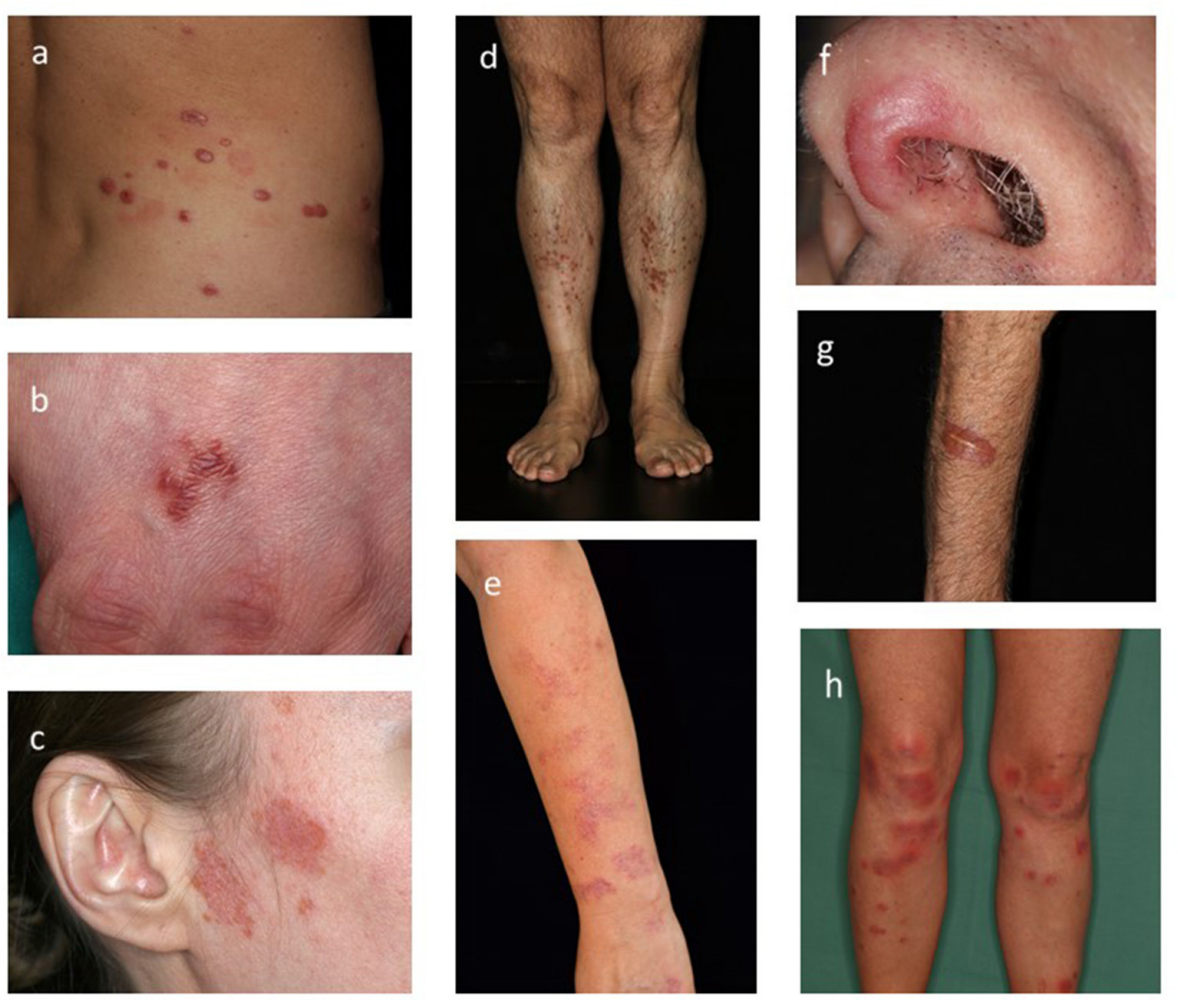
Clinical hallmarks of cutaneous sarcoidosis. **(a–g)** Specific sarcoid skin lesions. **(a)** Multiple violaceous nodules on the trunk. **(b)** Solitary red-brown plaques on the back of the hand. **(c)** red atrophic plaques with slight epidermal involvement. **(d)** Multiple small red-brown papules on the lower leg. **(e)** Bright red colored macules non the arm. **(f)** Violaceous plaque on the nose (Lupus pernio). **(g)** Red plaque associated to a scar. **(h)** Non-specific skin lesion: tender erythematous nodules with subcutaneous infiltration on the ventral lower leg (Erythema nodosum).

Sarcoidosis is a multi-system disease that may involve almost any organ system (1). Cutaneous sarcoidosis lesions may be the only clinical manifestation, yet patients with sarcoid skin lesions may have involvements of other organs, including the lungs, lymph nodes, eye, liver, heart, joints and brain (1–3). Cutaneous sarcoidosis can manifest with sarcoid specific skin lesions, these changes typically show granuloma formation in the tissue, non-specific sarcoid skin lesions on the contrary present histologically with inflammation in the absence of granulomas (1, 3). Clinically, the disease can present with red, red-brown or violaceous colored macules, papules, plaques or nodules (Figures 1a–g). There may be single or multiple lesions, which may exhibit epidermal involvement or prominent telangiectasia (4, 5). Lupus pernio (bluish-red or violaceous nodules/plaques over nose, cheeks and ears), subcutaneous nodules (Darier-Roussy disease), and scar- and tattoo associated sarcoidosis, are recognized clinical presentations (4, 5). Of note, all these described skin lesions are sarcoid specific, whereas the typical non-specific sarcoid skin manifestation is erythema nodosum. Characterstically, erythema nodosum, manifests as tender erythematous nodules with subcutaneous infiltration (Figure 1h), commonly on the ventral aspect of the lower leg, showing the histological findings of panniculitis with septal inflammation (1, 2). Given that sarcoidosis is a multisystem disease, the diagnosis requires involvement of at least two organs. Thus, whether skin-only sarcoidosis can be classified as sarcoidosis or should be termed sarcoid-like granulomatous disease remains controversial (1, 2).

The treatment of cutaneous lesions can be challenging, but the application of potent/superpotent topical steroids remains the mainstay of treatment (1, 2). For patients with severe or progressive skin lesions and/or widespread cutaneous involvement, systemic corticosteroids may be required (1, 2). In addition, steroid-sparing agents such as hydroxychloroquine and methotrexate are used (6, 7). Although randomized, controlled trials are lacking in cutaneous sarcoidosis, multiple reports suggest efficacy of agents such as azathioprine, tetracylcine, thalidomide, and infliximab (1, 2).

To date, large multi-center cohort data on clinical phenotypes and therapeutic responses in cutaneous-dominant sarcoidosis, remain scant (8–18). The aim of this study was to analyzed the clinical features and non-skin organ involvement in patients with cutaneous sarcoidosis.

## Methods

### Study population and definition of eligible cases

The current retrospective cohort study included all patients diagnosed with cutaneous sarcoidosis between January 1st, 2014, and December 31th, 2020 in the Department of Dermatology, University of Lubeck, Lubeck, Germany. The study was approved by the institutional ethical committee (21–292) in accordance with the Declaration of Helsinki. Written informed consent was obtained from all subjects. Cutaneous sarcoidosis was diagnosed based on compatible clinical manifestation in conjunction with typical histological features in all patients. Histopathological criteria for diagnosing cutaneous sarcoidosis were a granulomatous reaction pattern with discrete predominantly epithelioid granulomas without necrosis (non-caseating/naked granulomas), with few surrounding lymphocytes and often a rim of mild dermal fibrosis. Granuloma-forming infections (mycobacterial and fungal infections, leishmaniasis) as well as foreign body reactions, granulomatous rosacea, granuloma anunulare and granulomatosis with polyangiitis were exluded in all cases. Eligible patients were longitudinally followed from their diagnosis up to April 30th, 2021.

### Definition of covariates

The medical records of eligible patients were systematically reviewed, and the following variables were retrieved: clinical and morphological features, utilized therapeutic modalities (local/systemic treatment), co-morbidities and routine laboratory and radiologic findings at baseline. Impaired lung function was defined by pathological pulmonary function tests. Severe hilar lymphadenopathy was considered in the radiologic stages I-II of the disease (19).

Exposure to immune-modulating drugs was defined in patients managed by any of the following agents: systemic corticosteroids, doxycycline, azathioprine, methotrexate, hydroxychloroquine, and infliximab. Complete blood count parameters, C-reactive protein (CRP), angiotensin-converting enzyme (ACE), and soluble interleukin 2 receptor (sIL-2R) levels were assessed prior to the administration of any systemic therapy. The globally acceptable cutoffs were adopted to define leukocytosis, neutrophilia, thrombocytosis, and elevated CRP (20, 21). Elevated levels of ACE, sIL-2R, and calcium were defined beyond the cutoffs of 82 U/L, 623 kU/L, and 2.55 mmol/L, respectively.

### Statistical analysis

All continuous parameters were expressed as mean values (standard deviation [SD]). Percentages of different subgroups were compared by a Chi-square test. Normally distributed continuous variables were compared using the student *t*-test. To identify predictors of systemic involvement and acute disease course, a logistic regression model was employed to calculate odds ratios (ORs) and 95% (confidence intervals) CIs. SPSS software, version 25 (SPSS, Armonk, NY: IBM Corp), was used to perform all statistical analyses.

## Results

### Demographic and morphological features

Thirty-seven patients with cutaneous sarcoidosis were analyzed, of whom 21 (57%) were females and 16 (43%) were males. The mean (SD) and median (range) age of included patients was 52.2 (12.5) and 54.0 (22.0–77.0) years, respectively. The most frequently encountered manifestation of cutaneous sarcoidosis was papules (*n* = 16), whereas 9 patients displayed plaques and nodules. One patient had the subcutaneous variant (Darier-Roussy disease) and another had scar-associated sarcoidosis. Ten (27%) patients presented with erythema nodosum (Table 1). All patients were of the Caucasian ethnic group.

**Table 1:** Demographic and clinical characteristics of patients with cutaneous sarcoidosis.

**Age at diagnosis; years**	
Mean (SD)	52.2 (12.5)
Median (range)	54.0 (22.0–77.0)
**Sex, n (%)**	
Male	16 (43%)
Female	21 (57%)
**Morphology of cutaneous disease, n (%)** *	
***Specific cutaneous sarcoidosis***	
Papules	16 (43%)
Plaques and nodules	9 (24%)
Subcutaneous (Darier-Roussy disease)	1 (3%)
Scar-associated	1 (3%)
***Non-specific cutaneous sarcoidosis***	
Erythema nodosum	10 (27%)
**Localized involvement, n (%)**	14 (38%)
**Systemic involvement, n (%)**	23 (62%)
Bilateral hilar lymphadenopathy	23 (62%)
-Severe hilar lymphadenopathy (≥stage II)	6 (16%)
-Impaired lung function	11 (30%)
Arthritis	11 (30%)
Splenomegaly	6 (16%)
Uveitis	2 (5%)
Neurosarcoidosis	1 (3%)
Lofgren syndrome	4 (11%)
**Disease course, n (%)**	
Chronic	26 (70%)
Acute	11 (30%)
**Management, n (%)**	
Topical corticosteroids	24 (65%)
Systemic corticosteroids	12 (32%)
NSAID	5 (13%)
Hydroxychloroquine	2 (5%)
Methotrexate	1 (3%)
Azathioprine	1 (3%)
Doxycycline	1 (3%)
Infliximab	1 (3%)

n, number; SD, standard deviation; NSAID, non-steroidal anti-inflammatory drugs.

### Systemic involvement and disease course

While 14 (38%) patients had isolated cutaneous disease, 23 (62%) patients demonstrated a multi-systemic disease with at least two organs involved during follow-up. Of patients with systemic disease, the cutaneous disease followed, preceded, and coincided with the development of systemic sarcoidosis in in 2 (9%), 9 (39%), and 12 (52%) patients, respectively. Subgroup analysis of patients with preceding skin involvement showed that patients with isolated cutaneous sarcoidosis displayed another organ involvement after a median (rage) of 12 (3–84) months. Bilateral hilar lymphadenopathy was the leading systemic manifestation of the disease (***n*** = 23), followed by arthritis (***n*** = 11), splenomegaly (***n*** = 6), uveitis (***n*** = 2), and neurosarcoidosis (***n*** = 1). Eleven patients demonstrated impaired lung functions. Of note, stage I pulmonary sarcoidosis was present in 17 patients, stage II pulmonary sarcoidosis in 5 patients, and one patient was diagnosed with stage III pulmonary sarcoidosis. With regard to the disease course, 11 (30%) patients followed an acute course (sudden onset, non-recurrent course of disease), whereas 26 (70%) patients featured a chronic and indolent course (gradual disease course, often recurrent or progressive). Four Ten (27%) patients presented with Löfgren syndrome (Table 1).

### Management, laboratory analyses, and comorbidities

The vast majority of study participants (***n*** = 24; 65%) underwent topical corticosteroid treatment. Twelve (32%) patients were managed by systemic corticosteroids and six (16%) patients were placed on systemic immune-modulating drugs as delineated in Table 1. Five (13%) patients were treated by non-steroidal anti-inflammatory drugs (NSAIDs).

The laboratory analyses of eligible patients at baseline demonstrated in 21%, 76%, and 50% of patients increased levels of ACE, sIL-2R, and CRP, respectively. Less frequent laboratory findings included hypercalcemia (6%), leukocytosis (8%), neutrophilia (10%), and thrombocytosis (5%). Hypertension was present in 3 (8%) patients, whereas diabetes mellitus, asthma, and melanoma clustered with cutaneous sarcoidosis in two (5%) patients, each.

### Predictors of systemic involvement and acute disease course

Table 2 demonstrates a logistic regression analysis aiming to identify predictors of systemic involvement. Female sex (OR, 7.08; 95% CI, 1.60–31.33; *P* = 0.007) and younger age (<54 years; OR, 6.88; 95% CI, 1.48–32.01; *P* = 0.010) at disease onset were significantly associated with systemic manifestation. Acute disease course (OR, 1.92; 95% CI, 1.30–2.84; *P* = 0.002) were positive predictors of systemic involvement. Expectedly, elevated levels of ACE (OR, 1.44; 95% CI, 1.10–1.88; *P* = 0.049), sIL-2R (OR, 17.33; 95% CI, 1.39–216.60; *P* = 0.011), and CRP (OR, 11.00; 95% CI, 1.06-114.09; *P* = 0.025) also predicted the presence of systemic manifestation.

**Table 2:** Predictors of systemic involvement among patients with cutaneous sarcoidosis.

	**Patients with systemic involvement (*n =* 23)**	**Patients with exclusively cutaneous disease (*n =* 14)**	**OR (95% CI) [*P* value]**
Age < 54.0 years	15/23 (65%)	3/14 (21%)	**6.88 (1.48–32.01) [0.010]**
Female sex	17/23 (74%)	4/14 (29%)	**7.08 (1.60–31.33) [0.007]**
Acute disease course	11/23 (48%)	0/14	**1.92 (1.30–2.84) [0.002]**
***Morphological features***			
Plaques and nodules	4/23 (17%)	5/14 (36%)	0.38 (0.08–1.76) [0.208]
Papules	9/23 (39%)	8/14 (57%)	0.45 (0.08–2.67) [0.375]
***Laboratory measures***			
Elevated ACE	7/23 (30%)	0/10	**1.44 (1.10–1.88) [0.049]**
Elevated soluble IL-2 receptor	13/14 (93%)	3/7 (43%)	**17.33 (1.39–216.60) [0.011]**
Hypercalcemia	1/14 (7%)	0/4	1.08 (0.93–1.25) [0.582]
Elevated CRP	11/17 (65%)	1/7 (14%)	**11.00 (1.06–114.09) [0.025]**
***Management***			
Systemic corticosteroids	9/23 (39%)	0/14	**1.64 (1.18–2.28) [0.007]**
Topical corticosteroids	14/23 (61%)	14/14 (100%)	**0.61 (0.44–0.85) [0.007]**
NSAID	5/23 (22%)	0/14	1.28 (1.03–1.59) [0.061]
Immune-modulating drugs ^a^	5/23 (22%)	1/14 (7%)	3.61 (0.38–34.69) [0.243]

Bold: significant values.

OR, odds ratio; CI, confidence interval; CRP, C-reactive protein; ACE, angiotensin converting enzyme; IL-2, interleukin 2, NSAID, non-steroidal anti-inflammatory drugs.

^a^Doxycycline, azathioprine, methotrexate, hydroxychloroquine, or infliximab.

## Discussion

The most common clinical cutaneous manifestation in our cohort, were papular eruptions (43%). Comparing this data to cohort studies of different ethnic groups showed similiar results with papules (8, 9, 12, 16, 17) and plaques (11, 13, 14) being the most common skin features in cutaneous sarcoidosis. Our study demonstrated that sarcoid skin lesions were highly associated with additional systemic involvement. Specifically, 62% of the patients had systemic involvement. Of note, this is in line with previously published results (8). All our cutaneous sarcoidosis patients with systemic involvement showed bilateral hilar lymphadenopathy, of whom almost 50% also had an impaired lung function. Lung involvement in cutaneous sarcoidosis is frequent (8–10, 12, 13, 15–17, 22). The other organ manifestations were less pronounced, such as arthritis (30%), splenomegaly (16%) and uveitis (5%). Of note, the acute onset of erythema nodosum and/or periarticular inflammation or arthritis of the ankles, with bilateral hilar lymphadenopathy (and in some cases parenchymal infiltrates), accompanied by systemic symptoms such as fever and malaise (Löfgren's syndrome) represents systemic involvement (1, 2, 23, 24).

In more than half of the cases with systemic manifestation, cutaneous sarcoidosis occurred simultaneously with another organ involvement. Interestingly, nine patients developed a systemic disease after being diagnosed with an isolated cutaneous sarcoidosis. Indeed, organ involvement should be excluded in patients diagnosed with cutaneous sarcoidosis (5). Regular follow-up is also recommended to monitor disease activity and to allow early detection of systemic involvement in cases of sarcoidosis limited to the skin (1, 2). Expectedly, elevated serum levels of sIL-2R, ACE, and CRP predicted the presence of systemic manifestation (Table 2). It was already shown that sIL-2R is a sensitive biomarker for diagnosing sarcoidosis (and superior to ACE), moreover it was shown that elevated sIL-2R levels indicate systemic involvement (24). Furthermore, the correlation of sIL-2R levels and chest radiographic stage demonstrated that lower sIL-2R levels were present in patients without parenchymal lung disease, compared to those with parenchymal lung disease (25). This might be an explanation for the lower s-ILR levels in our study. Another possible explanation for the lower soluble IL-2R levels in patients with exclusively cutaneous sarcoidosis might be that patients with isolated skin lesions have a granulomatous inflammation pattern localized to the skin or no granulomatous reaction at all (non-specific sarcoid skin lesions), resulting in lower levels of sIL-2R. Further research, has to be performed to validate sIL-2R as a biomarker in isolated cutaneous sarcoidosis.

Hypercalcemia indicating a possible kidney involvement (26) or infrequently bone marrow sarcoidosis (27), was present in 6% of the tested patients. However, none of the patients in our cohort suffered from renal or bone marrow sarcoidosis. Furthermore, none of the patients showed endocrine or cardiac involvement (28). Of note, it was shown that the first-mentioned is under-diagnosed, and the latter is potentially occult and serious life-threatening, so patients should be proactively examined and investigated to exclude it (6).

In this analysis of Caucasians, cutaneous sarcoidosis was female-dominant (57%); this fact is in line with literature, most clinical studies suggest that cutaneous sarcoidosis is more common in women (8, 10–15, 17, 18). In addition, statistical analyzes showed that female patients and younger age of disease onset showed a higher tendency to additional organ involvement (Table 2).

Given the poor treatment response in localized cutaneous sarcoidosis, the frequent systemic involvement, and corticosteroids being still the mainstay of therapy, there is a pressing need for the development of new treatment strategies. Further research is required to the underlying cause of sarcoidosis and the path mechanisms driving the infiltration of non-caseating granulomas, leading to an often chronic and persistent inflammation.

Limitations of the study include the relatively small number of patients and the retrospective study design. This may have resulted in some degree of selection bias, given that the patients were attending a tertiary referral center.

In conclusion, this retrospective cohort study reveals that cutaneous sarcoidosis is frequently associated with additional systemic involvement, particularly when present in young females. Our findings underscore the importance of early recognition of cutaneous lesions of sarcoidosis, which may herald the development of systemic disease. Almost 25 % of patients displayed sarcoid skin lesions prior to systemic organ involvement.

## Data availability statement

The original contributions presented in the study are included in the article/supplementary material, further inquiries can be directed to the corresponding author/s.

## Ethics statement

The studies involving human participants were reviewed and approved by Institutional Ethical Committee of the University of Lübeck (#21-292). The patients/participants provided their written informed consent to participate in this study.

## Author contributions

KB: conception of the work, acquisition data, drafting the work, and writing manuscript. EL and DZ: critically revising, intellectual content, and final approval. RL: conception of the work, critically revising, and final approval. KK: data analysis, interpretation of data, critically revising, and final approval. All authors contributed to the article and approved the submitted version.

## Funding

This work has been supported by the Cluster of Excellence Precision Medicine in Chronic Inflammation (EXC 2167) from the Deutsche Forschungsgemeinschaft and the Schleswig-Holstein Excellence-Chair Program from the State of Schleswig Holstein.

## Conflict of interest

The authors declare that the research was conducted in the absence of any commercial or financial relationships that could be construed as a potential conflict of interest.

## Publisher's note

All claims expressed in this article are solely those of the authors and do not necessarily represent those of their affiliated organizations, or those of the publisher, the editors and the reviewers. Any product that may be evaluated in this article, or claim that may be made by its manufacturer, is not guaranteed or endorsed by the publisher.
